# Phosphides and Sulphides

**Published:** 1885-02

**Authors:** 


PHOSPHIDES AND SULPHIDES.

Messrs. Gale and Blocki (of Chicago), on account of the innumerable complaints of the phosphides and sulphides in pill form, have been led to investigate the cause.

Without entering into the details of their experimental processes, they make the general statement that the fault of these preparations is that they are pills.

"No phosphide or sulphide, especially sulphide of calcium and phosphide of zinc, should ever be dispensed in pill form, as it is a chemical impossibility to moisten either of these without setting free, and thus losing, the very element particularly desired, and to make pills for gelatine or sugar coating, it is absolutely necessary to use a moist excipient to get a mass."

"Our experiments convince us that the best method of administering these drugs is by triturating them with a small quantity of powdered elm bark and *capsulating dry*."

